# Forming Glasses from Se and Te

**DOI:** 10.3390/molecules14114337

**Published:** 2009-10-26

**Authors:** Bruno Bureau, Catherine Boussard-Pledel, Pierre Lucas, Xianghua Zhang, Jacques Lucas

**Affiliations:** 1 UMR 6226 Sciences Chimiques de Rennes – Verres & Céramiques, Université de Rennes 1–CNRS, Campus de Beaulieu, 35042 Rennes Cedex, France; 2 Department of Material Science and Engineering, University of Arizona, AZ 85721, USA

**Keywords:** selenium, tellurium, glass, infrared

## Abstract

Despite being close neighbors on the Periodic Table, selenium and tellurium present a totally different abilities to form glasses. Se is a very good glass former, and gives rise to numerous glass compositions which are popular for their transparency in the infrared range and their stability against crystallization. These glasses can be shaped into sophisticated optical devices such as optical fibers, planar guides or lenses. Nevertheless, their transparencies are limited at about 12 µm (depending on the thickness of the optical systems) due to the relatively small mass of the Se element. On the other hand, tellurium is heavier and its use in substitution for Se permits to shift the IR cutoff beyond 20 µm. However, the semimetallic nature of Te limits its glass formation ability and this glass family is known to be unstable and consequently has found application as phase change material in the Digital Versatile Disk (DVD) technology. In this paper, after a review of selenide glasses and their applications, it will be shown how, in a recent past, it has been possible to stabilize tellurium glasses by introducing new elements like Ga or I in their compositions.

## 1. Introduction

Despite being close neighbours on the Periodic Table and having similar electronic structure, Se and Te are totally opposite in terms of their ability to form glassy materials [1,2,3,4]. When synthesizing chalcogenide glasses the key chemical strategy is based on the formation of a polymeric melt exhibiting flexibility in the chemical bond angles which will result in the formation of a floppy network retaining strong chemical bonds but having no periodicity. When cooling down such a liquid the reorganisation of the skeleton is very slow and almost impossible when the temperature drops down and the viscosity increases. The final step of the process is the formation of a liquid with an infinite viscosity, a frozen liquid, in other word a glassy material. This situation is very rare in chemistry and leads to a solid with very specific properties which will be briefly reviewed here. Since a glass can be regarded as a super-cooled liquid, the resulting solid violates the elementary rules of thermodynamics and has to be regarded as a nonequilibrium solid. As a main consequence, when reheated, a glassy material keeps in memory its status of frozen liquid and is characterized by a glass transition temperature Tg. Above Tg the material relaxes, the viscosity is no more infinite and decreases with increasing temperature eventually leading to a viscous liquid. This exceptional property is used to shape the glass by moulding, blowing and fibering and is at the source of many industrial products such as windows, containers, optical fibres etc. These operations are not danger-free since thermal motion allows the viscous liquid to rearrange and to reoptimize its thermodynamic status typically to form crystallites which will transform the glass into a crystalline or a composite glass ceramic material.

Under ideal conditions, which means when this frozen inorganic polymer is free of defects such as bubbles or imperfections, it can be considered that a glass is the perfect material for light propagation. Indeed, the main reason for developing new glasses is associated to their optical properties. It is well known that the two intrinsic factors limiting the transparency of a glass are, first, the electronic absorption which affects the transmission in the UV visible or near IR region and second the phonon absorption due to the vibrational modes of the network which is prominent in the infrared region. The phonon absorption is directly related to the relative atomic mass of the elements used to build the glassy framework. Thus, the glasses made from heavy chalcogens such as Se or Te will offer a huge advantage for developing materials transparent in the infrared (See Figure 8).

The chemical challenge is then to develop infrared glasses resistant to crystallisation and being transparent as far as possible in the IR. The first strategic applications is related to the thermal imaging or infrared camera technology which requires optics transparent in the 8 µm to 12 µm region spanning the main optical window of the atmosphere as well as the black body emission of thermal objects at room temperature. The second application is associated to Fibre Evanescent Wave Spectroscopy which requires the development of optical fibres transparent in the window where the vibrational fingerprints of most molecules and biomolecules are located. Classical infrared spectroscopy shows that this region extends from 2 µm to 15 µm.

The main strategy for synthesizing new infrared glasses is based on the creation of a covalent polymeric framework involving elements having similar electro-negativity. This means that the central elements such as Se or Te have to be combined with close neighbour atoms in the Periodic Table such as As, Sb, Ge, Ga, or I. Before discussing the glass forming ability of these two chalcogens it is important to mention their significant differences when examined as individual solids.

**Figure 1:**
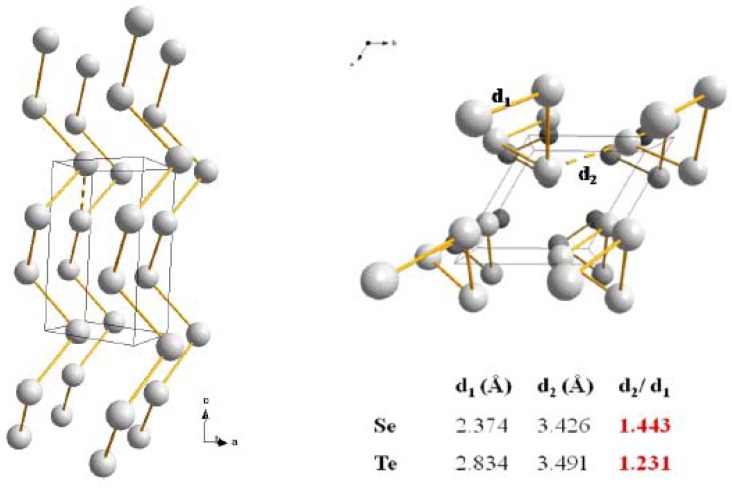
Hexagonal form of the Se and Te crystalline phases together with the relevant distances representatives of their opposite physical behaviors (see text) [6].

Both Se and Te belong to group 6 of the Periodic Chart and are characterized by an outer electronic shell of six electrons s^2^p_x_^2^p_y_^1^p_z_^1^. Only the two odd electrons are involved in the formation of strong covalent bonds to build the inorganic polymeric network. It is commonly assumed that the s electrons lie well below the p states and do not contribute to the bonding [5]. Indeed each Se and Te atoms can be viewed as generating a tetrahedron formed by the repulsion of two bonding electrons pairs and two nonbonding lone-pair electrons. As a result, the main structural building unit responsible for the formation of the solids is an infinite chain as represented in Figure 1, where the structures and bond lengths of the two solids are schematized. The main difference between Se and Te is the nature of the bonding responsible for the interchain cohesion. For Se the bonding is of Van der Walls origin and results from dipole/dipole interactions. This weak bond is easily destroyed by thermal agitation which is confirmed by a low melting point of T_m_ = 216 °C and the resulting formation of a viscous liquid. In the case of Te, the metallic character is much stronger as illustrated by a conductivity several orders of magnitude higher than for Se. Also the analysis of the interatomic distances unambiguously shows that a delocalized π bonding system reinforces the intrachains as well as the interchain cohesion of Te atoms. The results is in a higher melting point T_m_ = 422 °C and the formation of a more fluid liquid where atomic associations are very weak like in a molten metal.

Cooling down a Se melt, even with a moderate quenching rate, leads to a glassy material, while it is impossible to vitrify a bulk Te melt even with a severe quenching. Indeed a critical cooling rate of 1 C/s is enough to vitrify Se while an extremely fast quenching of 10^10^ C/s such as splash cooling is necessary to obtain small chips of vitreous Te. As a consequence, Se based glasses dominate the chemistry of chalcogenides glasses used for mid-IR applications. The sulphur based compounds are rather similar to the Se derivatives but have a moderate interest due to their limited transparency in the mid-IR. On the other hand the Te based glasses, due to their extreme tendency to crystallise have found applications as phase change materials for optical storage for example in the Digital Versatile Disk (DVD) technology [7,8,9].

## 2. Forming Infrared Transmitting Glasses from Selenium

It is clear that Se is a key element to form glasses having applications in the mid-IR. Its relative atomic mass M = 78.96 g·mol^–1^ is high enough to generate low energy vibration modes allowing transparency down to the 15–16 µm region. The two electronic lone pairs are well localised and the main bonding is covalent with a σ character permitting easy rotation and flexion. The lone pairs which play a steric effect introduce a nonbonding (nb) level in the energy diagram resulting in an optical band gap E_g_ between the nb level and the σ* antibonding level which is usually inferior to E_g_ = 2 eV. Consequently most of the Se based glasses have a black colour since they absorb visible light.

The main strategy to develop an IR glasses with potential applications is as follows: the glassy framework needs to have a strong nonperiodic character in order to avoid any structural recombination leading to microcrystals formation during the shaping process. This means that the first objective is to identify a glass composition highly resistant to devitrification. The same structural framework also needs to have a dimensionality compatible with acceptable mechanical and thermal properties. It is obvious that a chains-like framework will result in a poor rigidity with a low glass transition temperature. As an example the Se glass itself suffers from a T_g_ = 40 °C incompatible with practical applications. Consequently it is necessary to develop glass compositions having network dimensionality between 1D and 2D or 3D in order to ensure values of T_g_ superior to the arbitrary average value of 130 °C. In order to maintain the covalent character of the network while increasing the dimensionality, the 1D chains can be cross-linked using trivalent or tetravalent elements such as As, Sb, Ge having similar electro-negativities.

It must be noted that in the As/Se binary system the so-called “stoichiometric glass” As_2_Se_3_ correspond to the ideal 2D network resulting from the connection of distorted AsSe_3 _pyramids. The resulting planar network is floppy enough to provide a glassy material exhibiting good physical and rheological properties making this popular composition one of the most widely used. Comparatively, in the Ge/Se system the ideal 3D network based on the connection of GeSe_4 _tetrahedra is at the composition GeSe_2_. However, this composition leads to a network that is too rigid and results in poor glass forming ability, the best composition among this binary family is surely GeSe_4_ which is ideally seen as built up of tetrahedra linked to each other by Se-Se dymers, providing the degrees of structural freedom necessary to obtain an interesting material from a physical and thermo-mechanical point of view. Nevertheless, the actual structure of Ge/Se glasses is still the source of much debate and will be discussed below. Phillips and Thorpe have developed a general theory which predicts the network rigidity of chalcogenide glasses based on the average coordination number <r> defined as the average number of covalent bond per atoms [10,11,12]. It was shown that a network with a value of <r> = 2.4 is optimally constrained and displays optimum mechanical properties [11] as well as optimum glass-forming abilities [10].

**Figure 2:**
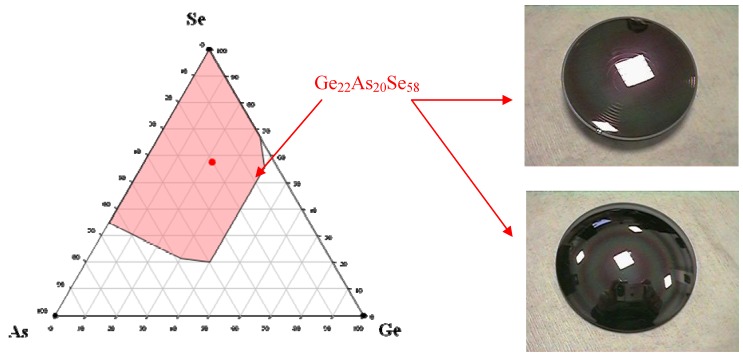
The Ge/As/Se system includes glasses with structural network dimensionalities between 2D and 3D. Their thermomechanical properties make them easy to be molded into sophisticated diffractive (above on the right) and aspheric (below on the right) lenses.

As observed in many glassy systems, the strategy to develop stable glasses versus crystallisation is to put into competition several crystalline species which delay the devitrification process. To achieve this goal, complex compositions are always preferred. For instance one of the industrial glass composition selected for moulding infrared complex optics is the so-called GASIR glass from the company UMICORE IR glass. Figure 2 represents the glass forming area in the Ge/As/Se system and the selected Ge_22_As_20_Se_58 _composition. In this glass the Se network is highly reticulated by the trivalent As and tetravalent Ge leading to a glass with a significant dimensionality and consequently a high T_g_ = 292 °C. When heated above T_g_ in the thermal regime where the materials become viscous, this glass does not show any crystallisation tendency and is therefore suitable for moulding optics under moderate pressure. Figure 2 shows some sophisticated infra red optics including aspheric and asphero-diffractive lenses which can be directly inserted into a thermal imaging system such as an infrared camera. The company UMICORE-IR produces such IR glass lenses for the automobile market and car manufacturers such as BMW or TOYOTA. The lenses are specially designed to equip systems for night vision driving assistance [13].

**Figure 3:**
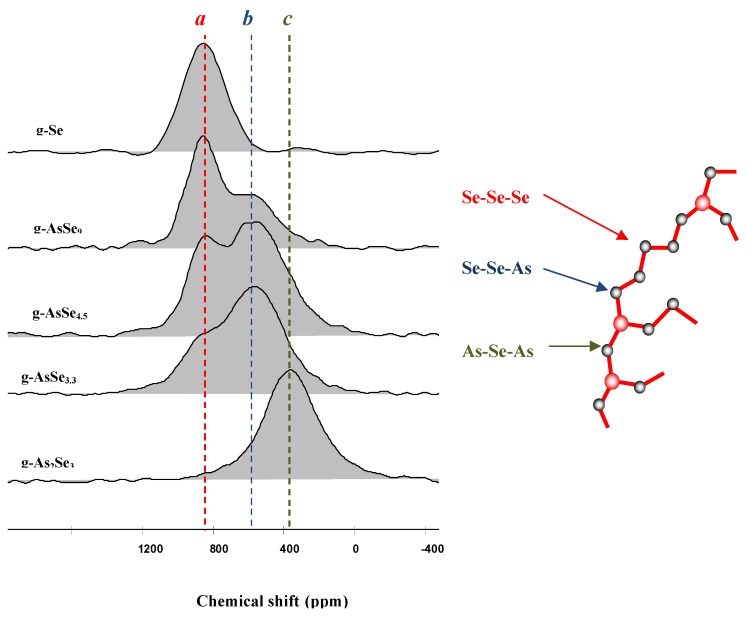
^77^Se solid state NMR spectra [17] in the As/Se system, showing the presence of three environments around the selenium: two selenium, two arsenic and one arsenic with one selenium. The NMR data are in good agreement with the expected classical model exhibiting a dimensionality between 1D (chains) and 2D (plan).

**Figure 4:**
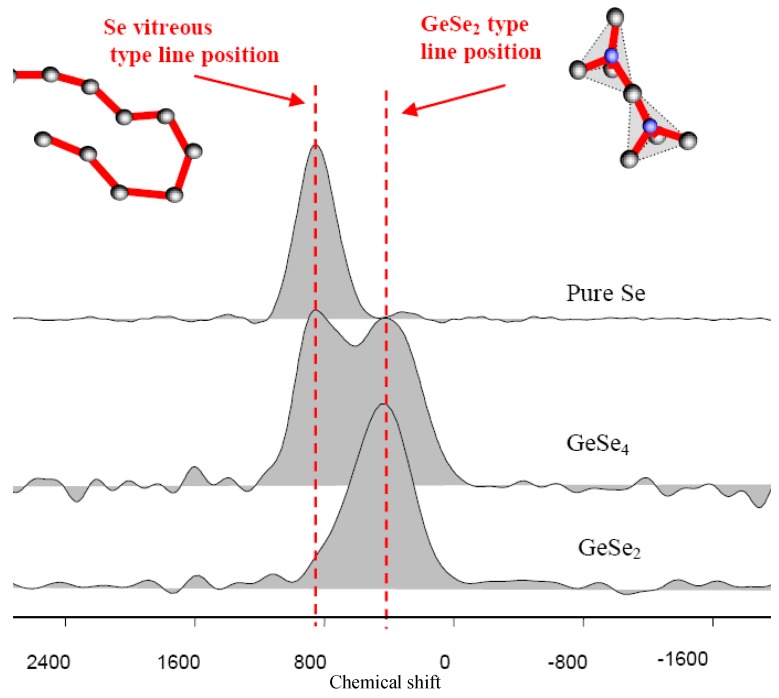
^77^Se solid state NMR spectra in the Ge/Se system, showing the presence of only two environments around the selenium : two selenium like in pure Se, and two germanium like in GeSe_2_.

From a more fundamental point of view, it is useful to relate the structure of chalcogenide glass to their physico-chemical properties. In that respect, it can be shown that ^77^Se is an interesting local probe for NMR investigation [14,15,16,17,18,19,20]. Since in disordered materials precise structural information from X-ray or neutron diffraction are difficult to obtain from pairs correlation functions, it is of prime interest to use solid state NMR to identify the selenium neighbourhood into the glass. For example, the NMR investigation of glasses formed along the Se (1D glass) to As_2_Se_3_ (2D glass) compositional range is very instructive. Figure 3 shows the variation of the ^77^Se NMR resonance signal ranging from Se atoms surrounded by two other Se atoms giving the local configuration Se-Se-Se of pure Se, down to the ultimate limit of As_2_Se_3 _where Se is surrounded by two As with the local environment As-Se-As. It is also seen that in an intermediary glass the signal is due to a Se-Se-As bonding. These NMR signatures allow to identify and count the number of different bonding configurations in a glass containing As and Se. The results are in very good agreement with the expected model described above [17]. On the other hand NMR investigations conducted on glasses formed in the Ge/Se system raise questions concerning the structural organisation of the glassy framework, in particular concerning the way the Ge atoms are cross-linking the Se chains. Figure 4 shows the NMR response of some glasses formed in this binary system. It must be mentioned here that several investigations indicates that GeSe_4 _is a singular composition corresponding to the optimisation of several properties such as resistance to crystallisation, fracture propagation etc. On the other hand the glass composition GeSe_2 _shows a severe tendency towards devitrification and cannot be a candidate for glass shaping such as fibre drawing, contrary to GeSe_4_ which is clearly the best glass in the system.

For the GeSe_2 _glass the parent crystalline material exists and is based on GeSe_4_ tetrahedra sharing corner and edges. It is reasonable to expect an overall similar structural connectivity in the liquid state and the glassy state which indicate that the chemical shift at 400 ppm in Figure 4 must be attributed to Ge-Se-Ge bonds. It is interesting to notice that the NMR results for the GeSe_4_ glass exhibit two lines; one precisely at 400 ppm and a strong one at 800 ppm characteristic of Se-Se-Se bonds as observed in pure Se [16]. These results are in agreement with Raman data which clearly shows two main vibrational modes for GeSe_4_, attributed to Se-Se (stretching mode) and to tetrahedra directly connected to each other (corner or edge shared) [21,22]. Hence, the initial intuitive model based on the 3D connection of GeSe_4_ tetrahedra connected by Se-Se pairs which implies unique bonding situation -Ge-***Se***-***Se***-Ge- is not consistent with those structural data and in particular with NMR results which clearly identify only two types of Se environments. Note that X-ray or Neutron diffraction techniques are not suitable to settle this point. Indeed they provide a Radial Distribution Function (RDF) with a first sharp maximum at 2.6 Å corresponding to Se-Se, Ge-Se or Ge-Ge first neighbour bonds, and a broader lines centred at 3.4 Å corresponding to second neighbours (Se or/and Ge) contained in chains, tetrahedra or connecting both [23]. Therefore, whatever the linking modes and the selenium chain lengths, the RDF presents the same shape.

To conclude on pure selenide glasses, it is interesting to note that while they are currently used to produce sophisticated industrial devices, a lot of work is still needed to fully understand their structural and physical behaviour.

## 3. Formation of Infrared Transmitting Glasses from Mixed Se/Te Compositions

**Figure 5:**
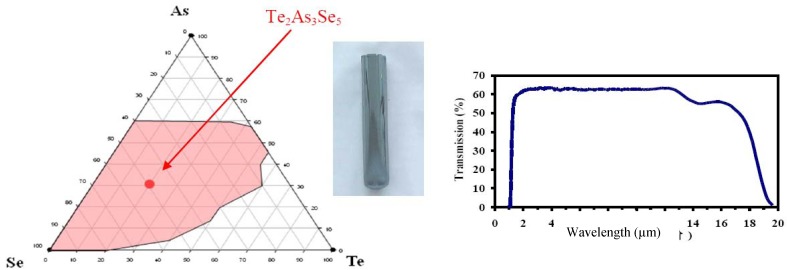
Te/As/Se system including the Te_2_As_3_Se_5_ composition (called TAS glass). The TAS can transmit IR light from 2 to 18 µm and do not exhibit any crystallization peak on DSC curve. This composition is easy to draw into fiber and constitutes an excellent candidate for remote infrared spectroscopy.

The search for glasses transmitting as far as possible in the infrared is motivated by the development of IR fibre optics having the largest optical window. Since the IR light must propagate through meters of fibers it is critical to decrease the optical loss in the low phonon region as much as possible. The best strategy is to increase the average relative atomic mass of the chalcogen components by using a Se/Te combination compatible with the glass stability. Indeed it is well known that using Te is beneficial for getting low vibrational modes, however it is also a risky operation because of the high tendency of Te to produce electron delocalisation and bonds prone to generate microcrystals [24]. The challenge is to develop a glass composition that maintains the Se character of the mixed chalcogens chains. In other words the Te atoms should be sufficiently separated such as to avoid electrons exchange by π bonding. This challenge has been overcome in the Se/Te/As ternary system represented in Figure 5 where the glass forming domain is depicted. The most stable glass composition free of any crystallisation peak is Te_20_As_30_Se_50_. In this so-called selected TAS glass the proportion of Se is more than twice the proportion of Te which allows a dilution of Te in the melt avoiding any Te-Te interactions. In this glass the mixed -Se–As-Se-Te-Se-As-Te-Se-As-Se- chains are cross-linked by As and form a ramified 2D structure. The rather low glass transition temperature Tg = 137 °C is compatible with this model. As shown in Figure 5, the TAS glass transmits IR light up to the multiphonon region located in the 18 µm region and due to its high stability towards crystallisation, it is a suitable candidate for drawing optical fibres from a glass rod. Additionally, it is possible to taper the optical fibre on-line in order to design shapes suitable for applications as optical sensors based on collecting the infrared signatures of molecules with IR fibres.

**Figure 6:**
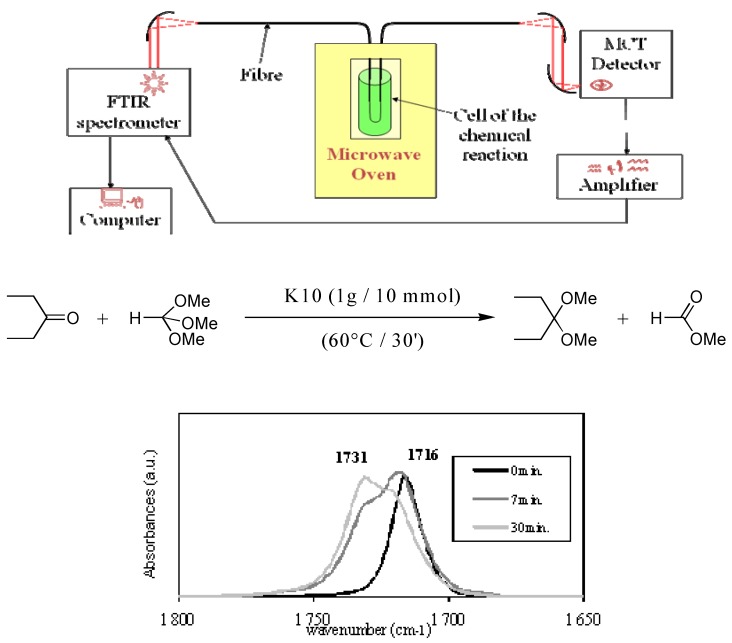
Experimental set up for FEWS carried out in microwave oven to follow an organic chemistry synthesis reaction.

Indeed the most promising and exciting application of TAS infrared glass fibres consists in collecting the infrared signature of any kind of molecules including biomolecules in-situ and in real time [25,26,27,28,29,30,31,32]. The principle of Fibre Evanescent Wave Spectroscopy (FEWS) is illustrated in Figure 6. It is well known that when IR light is confined into a fibre due to multiple reflections, part of the energy propagates along the surface of the fibre. If a molecule is put into contact with the fibre, this evanescent wave is absorbed by the vibrational modes of the molecule. Figure 6 depicts the IR source, the spectrometer, the fibre sensor and the detector of a FEWS system and shows that this remote analysis permits to record the infrared spectrum on any kind of molecules *in situ*. When the fibre is tapered along the sensing zone the reduction in diameter enhances the amplitude of the evanescent wave and consequently the sensitivity of the sensor. As an example among numerous applications, Figure 6 shows how FEWS was successfully implemented to follow an organic reaction under microwave irradiation [26]. The synthesis of 3,3-diethoxypentane by reaction of triethylorthoformate on 3-pentanone without any solvent has been studied *in situ*. The formation of the expected ketal 3,3- diethoxypentne and ethylformate has been followed by the growth of the C=O band at 1,731 cm^–1^ and the decrease of the C=O band at 1,716 cm^–1^.

Different type of investigations were devoted to the detection and growth of complex biomolecular system such as the colonisation of a surface by a bacteria, or the study of biological liquid (serum, blood) or tissues [33,34,35,36,37]. Indeed the medical field appears to be the most promising for the future of spectroscopy carried out with chalcogenide glass fiber. Ongoing works are in progress to design a prototype devoted to the detection of human pathologic state at an early stage.

## 4. Forming Glasses from Tellurium

A comprehensive review covering the formation of pure tellurium glasses has been published recently and can be consulted for further details [24]. Initially, Zhang and Lucas have reported the formation of glasses in binary systems such as the Te/Cl, Te/Br, and Te/I. These glasses exhibit interesting optical properties but also a low 1D dimensionality and consequently poor resistance to crystallization and weak mechanical and thermal properties. Hence their practical interest is very limited [38,39,40].

Other Te based glasses, especially glasses in the Te/Ge/Sb system, are known to be so unstable toward crystallization that they have found applications as phase change materials for optical storage, for instance in the Digital Versatile Disks, DVD technology. Under the impact of a laser beam having an adjustable power, very thin film materials can change reversibly from glass to crystal in a very short time. The reflectivity difference between the glassy and crystalline spots is sufficiently large to permit storage of binary information [7,8,9].

**Figure 7:**
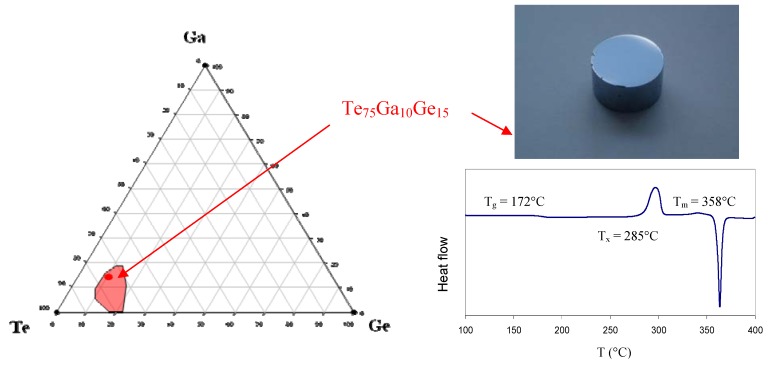
Te/Ge/Ga ternary system. These glasses do not contain any selenium and so are very sensitive to crystallization during the shaping as shown by the T_x _− T_g_ difference equal to 113 °C. On the other hand, they transmit the light over a wide range of wavelength until 25 µm depending on the thickness of the sample.

In the course of an investigation aiming at controlling the phase change it has been observed that the addition of a small amount of gallium Ga had a tremendous effect toward stabilizing the vitreousphase [41]. These research efforts are motivated by the recent interest expressed by some industrial or academic partners for stable optical devices transmitting light beyond 20 µm. For instance, the Darwin mission conducted by ESA, or its parent program from the NASA, requires a single mode fibre working from 4 µm to 20 µm. As a consequence it was decided to explore the Te/Ge/Ga system with the hope of discovering new stable optical glasses. Figure 7 shows the glass forming domain of the Te-Ge-Ga system. It must be noticed that the region where glasses can be obtained by moderate quenching is rather small and that the most stable composition is Te_75_Ge_15_Ga_10_. This so-called TGG glass is characterized by a value of T_x _− T_g_ = 113 °C which permit the preparation of bulk sample such as prism or lenses. Nevertheless the proximity of the crystallization to the softening temperature and the high enthalpy of the crystallisation peak as shown in Figure 7 make the preparation of optical fibres delicate.

**Figure 8:**
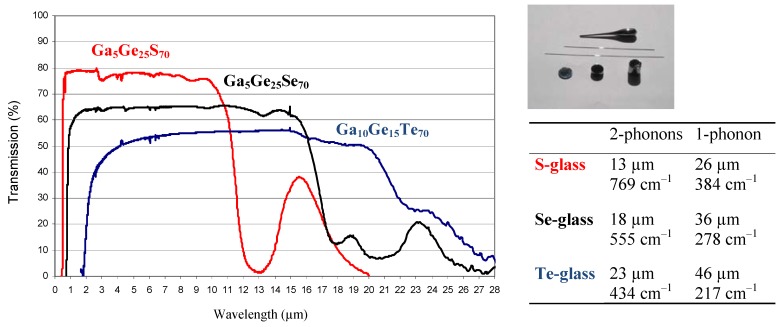
Transmission of tellurium glass (TGG) compared to selenide and sulfide equivalent glasses. They transmit the light over a wide range of wavelength until 25 µm depending on the thickness of the sample. TGI glasses exhibit an equivalent optical transmission and are easier to shape into optical fibers. More generally, The IR cut off is due to the multi phonon absorptions related to the relative weight of S, Se and Te respectively.

Another strategy used to prepare glasses from tellurium takes advantage of the fact that a monovalent atom such as iodine plays the role of terminal atom and gives some flexibility to the network in order to help glass formation [42]. In addition iodine atoms tend to trap the conducting electrons from tellurium helping prevent Te from crystallising. Experiments indeed demonstrate that iodine play a key role in the ability of Te to form glasses. For instance, the glass Te_73_Ge_20_I_7_, in the middle of the diagram, is characterized by a value T_x _− T_g_ = 124 °C which makes this glassy material suitable for moulding and even fibering. As discussed above, the main interest of these new vitreous materials is to obtain a very broad optical window extending from 2 µm to more than 20 µm, the largest ever obtained in a glass. The transmission spectra of the TGG and TGI glasses are very similar and are presented in Figure 8. It must be mentioned that while the presence of iodine is beneficial for stabilizing the glass it also tends to generate molecular volatile species when the glass is in the fluidic regime above Tg. This behaviour is a handicap for drawing fibres.

Finally, a third strategy consists in adding a small amount of Se to help stabilize the GeTe_4_ composition against crystallization while keeping the optimal window of pure Te glass [43]. The Te/Ge/Se system was investigated in the Se poor region and it was demonstrated that a few percent of Te substitution by Se around the GeTe_4_ composition was very beneficial for increasing the glass forming ability. As an example the glass Te_77_Ge_20_Se_3_ is characterized by a value of Tx − Tg = 115 °C which is compatible with fibre drawing. Nevertheless the presence of only 3% of Se in the glassy framework is penalized in the IR region by a small absorption shoulder around 18µm.

## 5. Concluding Remarks

The development of infrared technologies such as night vision cameras or bio-optical sensors is driving the search for new amorphous materials that combine unprecedented wide optical window with optimal rheological properties. The ability to fiber or mold these glasses enables manufacturing of optical components at a reasonable price and is the key for their commercial development. Multiple strategies can be adopted to design a glass with good stability and appropriate optical properties. Se based glass are intrinsically good glass formers but are limited in transparency in the long wavelength regime. Te glasses on the other hand are poor glass formers but possess very large optical windows. A glass based on a mix of Se and Te can then lead to a good balance between the required properties but ultimately, the low phonons intrinsic to Se vibrations limit the optical window, or the metallic nature of Te limits the glass formation ability. Possible solutions involve modifying the glassy network with heavy element that can improve the glass forming ability without detrimental effect on the optical window. Iodine was shown to play this role but present other manufacturing issues. The search for the perfect IR glass is therefore still on-going and remains the subject of much investigation.
